# Mr. Blegborough, on Chronic Croup

**Published:** 1806-06

**Authors:** Henry Blegborough

**Affiliations:** Dean Street, Finsbury Square


### *To the Editors of the Medical and Phyſical Journal.*

Gentlemen,

In the progress of Society from rude barbarity to refined luxury, not only the phenomena of well known diseases are greatly varied, but fresh combinations of symptoms, and even now and then new maladies, shew themselves. A principal purport of your very useful publication, I apprehend, to be that of recording these varieties and changes;

 and and should there be any thing in these remarks worthy attention, I need not solicit their insertion.

I have noticed, lately in particular, a disease affecting children, which is, so far as I know, non-descript. In my enquiries amongst medical friends, I have found very few who have seen, or at least given attention to it; some, however, have noticed it, and their observation of its symptoms and unhappy termination correspond but too exactly with my own. For these reasons I have been induced to attempt a description of this disease, to recount the means I have employed for its relief, with their effects, or rather perhaps with their inefficacy, hoping that some of your learned and ingenious Correspondents, having been more successful in their treatment, may be induced to unfold to the public the *means* of their success.

One of my friends has called this disease, the principal feature of which seems to be a spasmodic affection of the glottis, Chronic croup. As terms are of little consequence, provided their meaning be defined, and as in some of its symptoms it does a little resemble croup, I shall adopt the term.

The cases which have fallen under my observation have happened to children from three months old to two years.

The disease commences with a slight degree of strangling and hooping, generally first noticed on swallowing liquids, not exciting cough as if some part thereof had passed into the trachea, but a spasmodic action of the glottis and muscles of the larynx, as I have said, slight at first, but in the progress of the disease so complete as to resist the passage of air into the lungs for a considerable time; at length, however, it rushes in with a deep sonorous hoop, exactly resembling that of chin-cough, but no cough attends. This hooping or crouping is continued for a number of times, gradually abating in force, when the child losing its turgid countenance and difficult breathing, recovers its spirits and appears in perfect health. Soon, however, the irritation and strangling become so great as to cause convulsions, more or less strong; this sometimes happens in every attack, generally however, not more than once or twice a day, though the hooping may recur every hour. Now the child becomes emaciated. During the convulsions the hands and feet are not merely contracted but drawn into a peculiar cup-like form, which they never, even though the fits be for many days suspended, completely lose.

Soon after, the strangling on drinking takes place; the same same is observed on the child awaking; or, the child is always awoke with a sense of strangling and with hooping. By the bye, it may be observed here, that some children are affected in this way on awaking, without ever having any other symptoms of the disease; and this, so far as I have had opportunity of observing, is unattended with danger.

When the disease has subsisted some days, there is generally thick and short breathing, with heat of skin and frequent pulse; but as these symptoms are always relieved by a calomel purge, I conclude they are produced by loaden bowels. Being removed, they always in a few days return, and are, by the same means, again and again relieved.

These symptoms run on with greater or less violence from one month (the shortest period at which I have seen death supervene) to three, (clearly distinguishing this affection from inflammatory croup). At length they all suddenly become worse; the fits return with redoubled violence and frequency, death soon closing the scene.

So far as my observations go, this disease is not infectious.

Children at the breast, with others, are attacked indifferently.

For its cure I have nothing efficient to offer.

I have tried the whole class of anti-spasmodics in vain, except that the external application of æther seemed, on one occasion, to afford temporary relief; (was it from abstraction of heat by evaporation?) and that small doses of laudanum, with blistering of the throat and chest, and the calomel purge mentioned above, have, in all the cases I have seen, produced a very considerable abatement of symptoms, often entirely suspending convulsion for days together; but in the end they have uniformly returned, with horrid aggravation, hurrying on the sad catastrophe.

Bark, when the disease had continued for some time, appeared serviceable on one occasion.

Leeches never produced benefit.

Digitalis never did good.

Emetics always seemed useless.

The warm-bath sometimes abated violence of convulsion.

I have not tried cicuta.

The symptoms never warranted general bleeding.

I regret, deeply regret, that the amiable but mistaken affection of mothers has always prevented the examination of the parts after death.

In the anxious hope of obtaining some more scientific account of the nature of this disease, as well as a more effectual mode of treatment, by means of your valuable Journal,

I am, &c.

*Dean Street, Finsbury Square, April 15, 1806.* HENRY BLEGBOROUGH

